# Genetic characterization of hypervirulent *Klebsiella pneumoniae* responsible for acute death in captive marmosets

**DOI:** 10.3389/fvets.2022.940912

**Published:** 2022-08-04

**Authors:** Komkiew Pinpimai, Wijit Banlunara, Wendi D. Roe, Keren Dittmer, Patrick J. Biggs, Rachod Tantilertcharoen, Katriya Chankow, Napawan Bunpapong, Pongthai Boonkam, Nopadon Pirarat

**Affiliations:** ^1^Aquatic Resources Research Institute, Chulalongkorn University, Bangkok, Thailand; ^2^Department of Pathology, Faculty of Veterinary Science, Chulalongkorn University, Bangkok, Thailand; ^3^School of Veterinary Science, Massey University, Palmerston North, New Zealand; ^4^Veterinary Diagnostic Laboratory, Faculty of Veterinary Science, Chulalongkorn University, Bangkok, Thailand; ^5^Center of Excellence for Emerging and Re-Emerging Diseases in Animals, Chulalongkorn University, Bangkok, Thailand; ^6^Wildlife Exotic and Aquatic Pathology Research Unit (WEAP RU), Department of Pathology, Faculty of Veterinary Science, Chulalongkorn University, Bangkok, Thailand

**Keywords:** *Klebsiella pneumoniae*, marmoset, human, hypervirulent, K2, ST65

## Abstract

*Klebsiella pneumoniae* is a Gram-negative bacterium implicated as the causative pathogen in several medical health issues with different strains causing different pathologies including pneumonia, bloodstream infections, meningitis and infections from wounds or surgery. In this study, four captive African marmosets housed in Thailand were found dead. Necropsy and histology revealed congestion of hearts, kidneys and adrenal glands. Twenty-four bacterial isolates were obtained from these four animals with all isolates yielding identical phenotypes indicative of *K. pneumoniae* based on classical identification schema. All the isolates show the susceptibility to amikacin, cephalexin, doxycycline, gentamicin, and enrofloxacin with intermediate susceptibility to amoxycillin/clavulanic acid. One isolate (20P167W) was chosen for genome analysis and determined to belong to sequence type 65 (ST65). The genome of 20P167W possessed multiple virulence genes including *mrk* gene cluster and *iro* and *iuc* gene cluster (salmochelin and aerobactin, respectively) as well as multiple antibiotic resistance genes including *bla*_*SHV*−67_, *bla*_*SHV*−11_, *oqxA, oqxB*, and *fosA* genes resembling those found in human isolates; this isolate has a close genetic relationship with isolates from humans in Ireland, but not from Thailand and California sea lions. Phylogenetic studies using SNP show that there was no relation between genetic and geographic distributions of all known strains typing ST65, suggesting that ST65 strains may spread worldwide through multiple international transmission events rather than by local expansions in humans and/or animals. We also predict that *K. pneumoniae* ST65 has an ability to acquire genetic mobile element from other bacteria, which would allow Klebsiella to become an even greater public health concern.

## Introduction

*K. pneumoniae* is a gram-negative bacterium that can be found in environments such as soil, surface water, sewage, plants, also on the surface of medical equipment (1–3). *K. pneumoniae* can also be found as part of the natural flora on mucosal surfaces of mammals i.e., the gastro-intestinal and respiratory tracts. *K. pneumoniae* is also a causative agent associated with a wide range of infections in humans and animals (1). At present, two forms of *K. pneumoniae* infection have been described: (1); *K. pneumoniae* (classical strains) acts as an opportunistic pathogen causing infection in immunocompromised patients and (2); *K. pneumoniae* (hypervirulent strain (HV)) is a pathogen that can infect healthy people, causing mainly liver abscesses with the ability to spread to multiple organs (3). Most of the isolates from invasive infections have a characteristic hypermucoviscous (HMV) phenotype (3, 4). Initially, the hypermucoviscous phenotype was thought to be a characteristic of hypervirulent strains (5). However, not all hypermucoviscous phenotypes are hypervirulent and in the same way, not all hypervirulent strains have the hypermucoviscous phenotype (1, 6). Later on, HV *K. pneumoniae* are found to possesses multiple virulence factors which are inconsistent in each isolate, including multiple siderophore associated genes, *rmpA/A2 (*regulator of the mucoid phenotype), *peg-344* (a transporter located on the inner membrane), *allS* (associated with allantoin metabolism)*, kfu* (gene encoding an iron uptake system) and *mrkD* (type 3 fimbrial adhesion) (7, 8).

HMV *K. pneumoniae* has also been reported in animals such as New Zealand sea lions (9, 10), California sea lions (11, 12), African green monkeys (13, 14), and marmosets (15). Some of these infections were caused by HV *K. pneumoniae* (10, 15). These animal-based infections are a concern for human and veterinary health, in particular, when the infections occur in animals that are in close contact with humans. Here we characterized the genome of hypermucoviscous *K. pneumoniae* ST 65 isolated from a captive marmoset compared with other *K. pneumoniae* ST 65 in human and California sea lion isolates, which are serious public health concerns.

## Materials and methods

### History, gross, and histology exams

The marmosets (23 animals) were imported from Africa 2 years prior. They were raised as couples or individually in open cages at a private house in Bangkok. A total of four marmosets (two females and two males) were found dead with salivary foam in the mouth. There was no history of previous sickness.

After this event the living area of marmosets was clean up with disinfectant. The rest of the animals were kept in the same place. The was no clinical signs before and after this event as well as human that closed related with these animals. The deceased animals were stored at −20°C before being sent for necropsy. Tissue samples collected at necropsy from all marmosets included lung, liver, brain, kidney, lymph node and spleen. The tissues were separated into two sets; one was kept in 10% neutral buffered formalin for routine histopathology, and the other set was fresh tissues for microbial and viral analysis. For histopathology, the fixed tissues were embedded in paraffin, and followed the routine histology process. The 4 μm tissue sections were stained with hematoxylin and eosin (H&E) and Brown and Brenn to visualize bacteria.

### Disease screening and bacterial identification

Lung samples from all marmosets (*n* = 4) were sent to the Center of Emerging and Re-emerging Infectious Disease on Animals (EIDAs), Chulalongkorn University to screen for SARS-CoV-2. The organs including lung, liver, brain, kidney, lymph node and spleen of the 4 animals were inoculated on blood agar and incubated at 37°C in aerobic conditions overnight. If a single colony-type or a mixed culture with a predominance (>80%) of one colony type was seen, the colony was sub-cultured onto blood agar for further identification using matrix-assisted laser desorption ionization–time of flight mass spectrometry (MALDI-TOF) (16).

### String test, capsular serotype, *rmpA* gene identification

All the bacteria (*n* = 24) isolated from the tissues from four animals were identified as *Klebsiella pneumoniae*. The bacteria were further characterized for string test, capsular serotype and *rmpA* gene using polymerase chain reaction (PCR). String test was done using the following protocol. A standard bacteriological loop was used to stretch a viscous string from the colony. A positive string test was designated when the formation of viscous strings extended vertically for more than 5 mm (4).

DNA of each isolates were extracted using the PureLink^®^ Genomic DNA Mini Kit (Invitrogen, CA, US) and DNA from all *K. pneumoniae* isolates were used to identify the capsular serotype and presence of *rmpA* gene by PCR using primers in Table 1.

**Table 1:** Primer information for each target gene.

**Target**	**Primer**	**Sequence (5^′^−3^′^)**	**Product** **size (bp)**	**Reference**
Capsular type K1	*magA* F	GGTGCTCTTTACATCATTGC	1,283	(4)
	*magA* R	GCAATGGCCATTTGCGTTAG		
Capsular type K2	*K2wzy* F	GACCCGATATTCATACTTGACAGAG	641	(17)
	*K2wzy* R	CCTGAAGTAAAATCGTAAATAGATGGC		
*RmpA*	*rmp*A F	ACTGGGCTACCTCTGCTTCA	516	(18)
	*rmp*A R	CTTGCATGAGCCATCTTTCA		

### Antimicrobial susceptibility

The antimicrobial susceptibility in all isolates (*n* = 24) were tested according to the CLSI (Clinical and Laboratory Standard Institute) M02-A12 protocol using six antimicrobial agents: amoxycillin/clavulanic acid, amikacin, cephalexin, doxycycline, gentamicin, and enrofloxacin.

### Genomic DNA preparation and whole genome sequencing

*K. pneumoniae* isolated from the lung of marmoset No. 1 (20P167W) was subjected to whole genome sequencing. The PureLink^®^ Genomic DNA Mini Kit (Invitrogen, CA, US) was used to extract genome-quality DNA from a single colony cultured on blood agar, which was sent to Macrogen, Inc (Seoul, South Korea) for whole genome sequencing. A fragment library was prepared using a TruSeq^®^ Nano DNA library preparation kit (Illumina, Inc., San Diego, CA, US). Paired-end reads (2 X 100 bp) were obtained from a HiSeq instrument (Illumina, Inc., San Diego, CA, US). FastQC (https://www.bioinformatics.babraham.ac.uk/projects/fastqc/) was used to check the quality of the data. The de novo assembly was done using SPAdes in careful mode (version 3.14.1) (19). QUAST (version 4.5) (20) was used to evaluate the genome assemblies and determine their GC content. The contigs produced from SPAdes were annotated by Prokka using the default parameters (version 1.14.5) (21).

### Multilocus sequencing typing (MLST)

The genome in FASTA file were uploaded to Bacterial Isolate Genome Sequence Database (BIGSdb) servers (https://bigsdb.web.pasteur.fr/klebsiella/klebsiella.html), to analyze the sequence type. Sequence homology matching was performed using BLAST (22) with a word size of 15 and identity of 70% for DNA sequences. The *K. pneumoniae* MLST scheme used the following seven housekeeping genes (23): *gapA, infB, mdh, pgi, phoE, rpoB, and tonB*.

### Pan and core genome

The MLST result of the marmoset isolation was ST 65, and 36 other publicly available *K. pneumonia* ST65 genome data were retrieved from GenBank/EMBL (Supplement) for future study. Roary (version 3.9.1) (24) was also used to calculate the pan-genome (25) to identify the core and accessory genes by using annotated assemblies in GFF3 format previously produced by Prokka (21).

### Virulence and antimicrobial resistance genes

The whole genome data was used to analyze virulence genes using Kleborate (26) and the virulence factor database (VFDB) (27). For the antimicrobial resistance gene, the whole genome data was analyzed with ResFinder 3.0 (28) on the Center for Genomic Epidemiology servers (http://www.genomicepidemiology.org/).

### Phylogenetic tree

We used Snippy version 4.6 (https://github.com/tseemann/snippy) to identify core single-nucleotide polymorphisms (SNPs) using GCA_008572845.1_Singapore as a reference. The results were used to construct a phylogenetic tree using Geneious tree builder (V9.1.8) and visualized using SplitsTree4 (V4.17.0).

## Results

### Gross and histology exams

**Figure 1:**
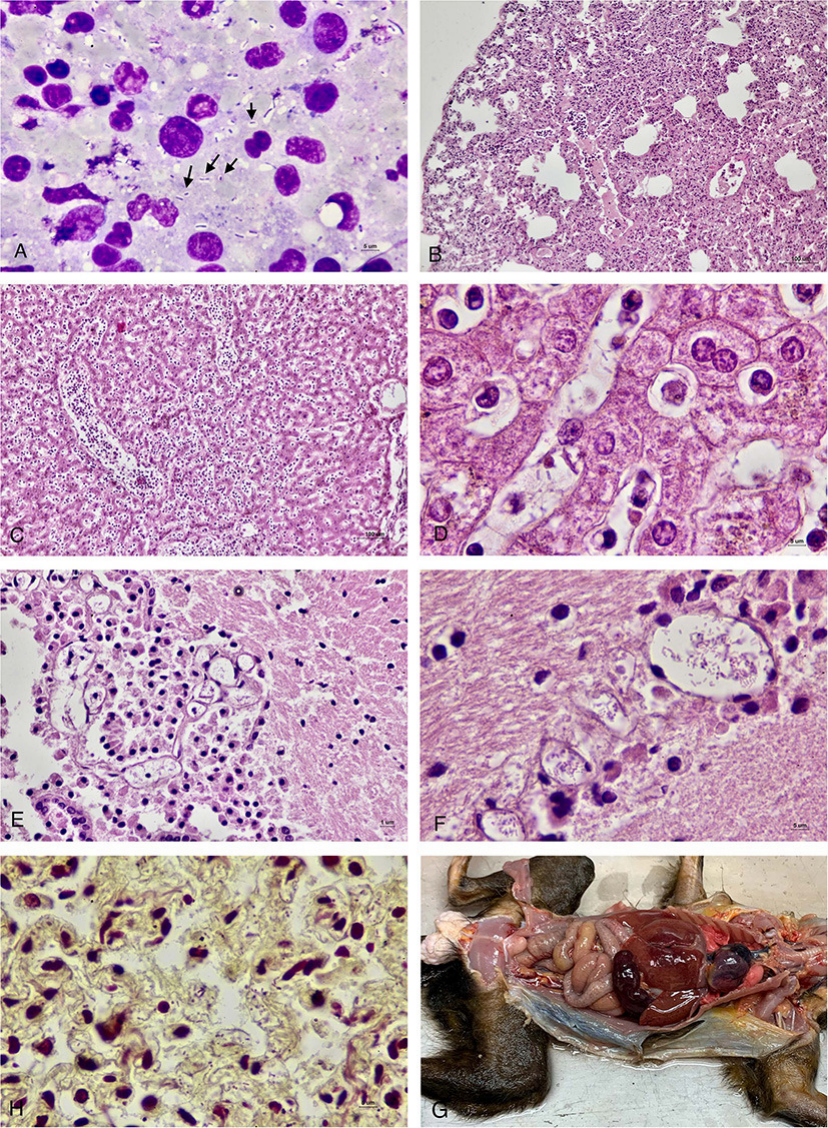
**(A)** Impression smear of the lymph node of marmoset No 4. showed numerous rod-shaped bacteria (arrow) along with neutrophils and lymphocytes. **(B)** Lung of marmoset No. 1 with mild to moderate suppurative pneumonia with pulmonary edema and emphysema. **(C)** Liver of marmoset No. 1 showed suppurative hepatitis. **(D)** Liver of marmoset No. 1 with rod-shaped bacteria in the sinusoids. **(E)** Brain tissue of marmoset No. 2 showed rod-shaped bacteria in the blood vessel. **(F)** Brain tissue of marmoset No. 1 with numerous rod-shaped bacteria in the blood vessels. **(G)** Lung of marmoset No. 2 with Gram-negative rod-shaped bacteria in the alveolar capillaries (Brown and Brenn). **(H)** Gross picture of marmoset No. 1.

On gross examination, all four animals had multilobular pneumonia. Congestion was seen in other organs including hearts, kidneys and adrenal glands. In marmoset No. 1 and No. 2, the spleens were enlarged and congested. There were no gross lesions in the brain, spinal cord or eyes in any of the animals. Microscopically, there was marked lymphoid necrosis with rod-shaped bacteria, marked suppurative splenitis and mild hepatitis. The lungs had mild suppurative pneumonia with pulmonary edema, emphysema and extramedullary hematopoiesis. Occasionally, rod-shaped bacteria were seen in alveoli (Figure 1). Rod-shaped bacteria also were seen in other organs including liver, brain, and lymph nodes.

### Disease screening, bacterial identification, string test, capsular serotype, and *rmpA* gene

All of the lung tissues were negative for SARS-CoV-2. All of the tissue samples including the lung, liver, brain, kidney, lymph node and spleen of four the animals were positive for *K. pneumoniae* by bacterial culture. The bacteria species was confirmed by MALDI-TOF (16). All *K. pneumoniae* colonies were round, mucoid and 4.2 ± 0.3 mm in diameter and gave a positive string test. All the isolates (*n* = 24) were positive for *rmpA* and positive for *K2wzy* (serotype K2).

### Antimicrobial susceptibility

All isolates (*n* = 24) were susceptible to five antimicrobial agents; the exception was amoxycillin/clavulanic acid, which showed intermediate susceptibility.

### *Klebsiella pneumoniae* genome feature

The *Klebsiella pneumoniae* genome was 5,342,477 bp in size with GC(%) 57.25. The whole-genome data was deposited in GenBank under project ID PRJNA664586 with accession numbers JAGLAL000000000 / SRR12678423. Using Prokka, there were 4,956 CDS, 3 rRNA, 68 tRNA, and 1 tmRNA. The *Klebsiella pneumoniae* isolated from the marmosets was identified as capsular serotype K2 sequence type 65 (ST65) by using Bacterial Isolate Genome Sequence Database (BIGSdb) servers.

### Pan and core genome of ST65

**Figure 2:**
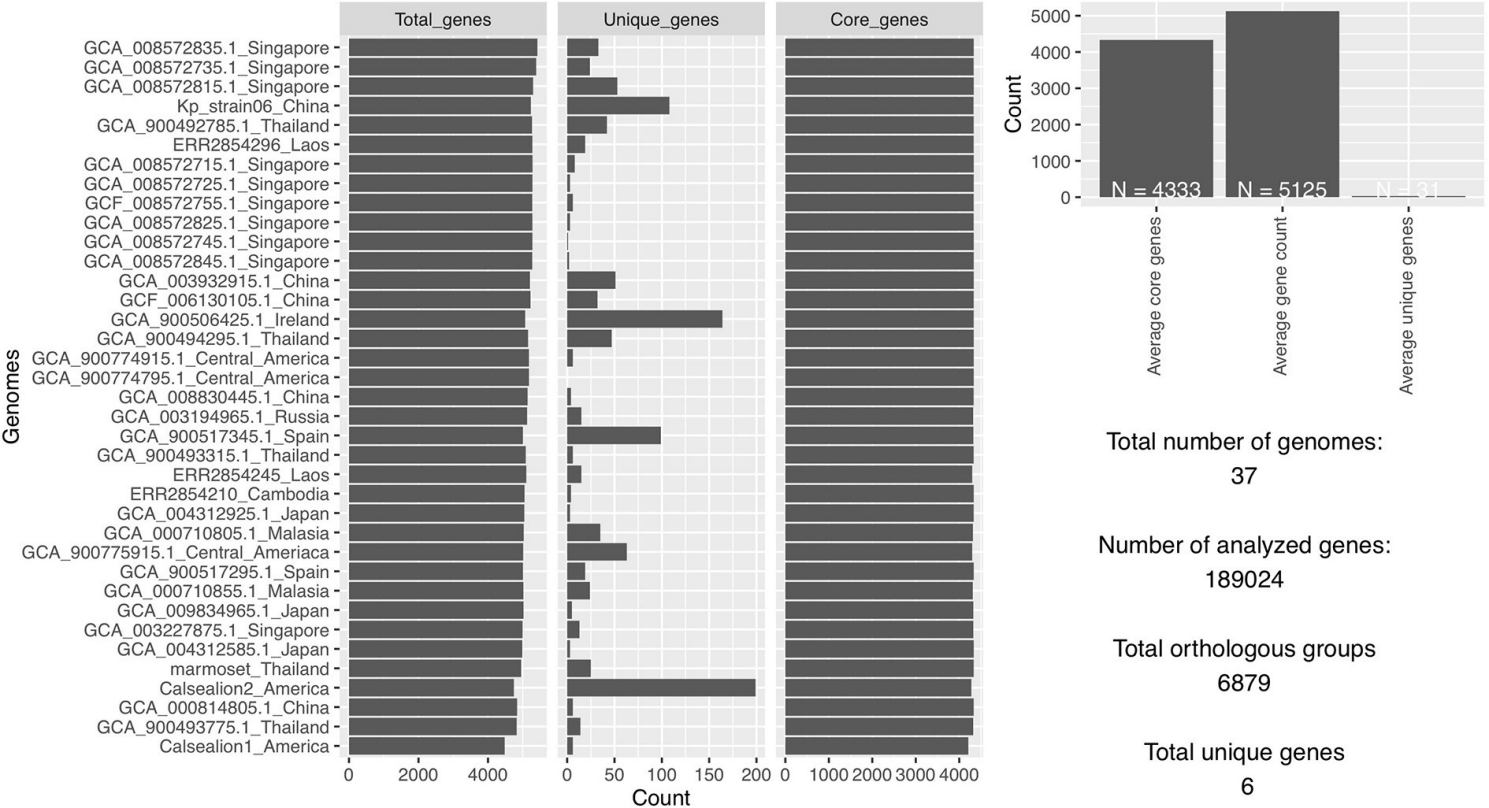
The total number of analyzed genes from 37 *Klebsiella pneumoniae* genomes was 1,89,024 of which 6,879 were in orthologous groups and 6 were unique genes.

**Figure 3:**
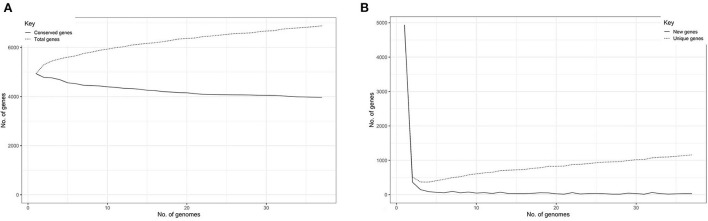
**(A)** Graph illustrating the decrease in number of conserved genes correlating with the increase of sample size. **(B)** Graph illustrating the increase of unique genes correlating with the increase of sample size.

The total number of gene clusters was 6,879: cloud = 1,543 (0% < = strains <15%), shell = 1,003 (15% < = strains <95%), soft core = 363 (95% < = strains <99%) and core = 3,720 (99% < = strains < = 100%). The total number of core genes and accessory genes of each isolate are shown in Figure 2. The conserved genes decreased when more genomes were added. In contrast, the number of unique genes increased when more genomes were added (Figure 3).

### Virulence and antimicrobial resistance genes

The virulence gene, the ST65, showed both similarity and difference. All of ST65 isolates possessed type I and III fimbriae-associated genes (*fim* and *mrk*). They possessed variation of siderophore associated genes (Aerobactin, Salmochelin and Yersiniabactin). Some of them possessed Colibactin genotoxic metabolite causing DNA damage. For antimicrobial resistant genes all the isolates possess *bla*_*SHV*−67_, *bla*_*SHV*−11_, and *fosA* genes. Some human isolates also possessed other antimicrobial resistance genes, which differed in each isolate.

**Figure 4:**
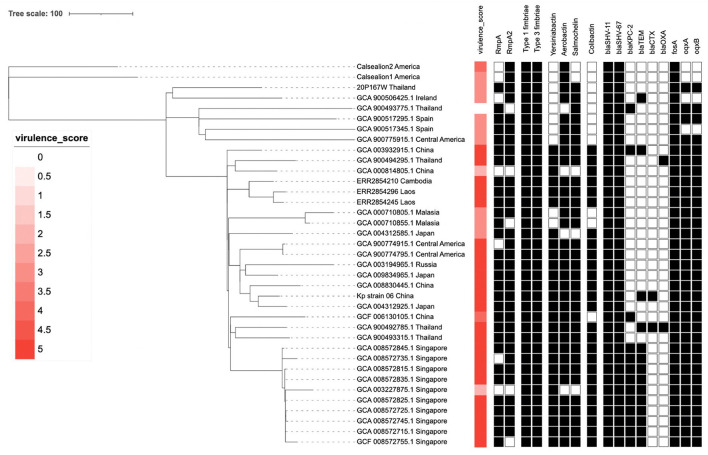
The phylogenetic tree of 37 *Klebsiella pneumoniae* ST65 built from publicly available plus one isolate from this study. The tree was based on core single-nucleotide polymorphisms. The marmoset isolate from Thailand (20P167W) was grouped together with the isolate from Ireland.

The Kleborate platform revealed 3/5 virulence score in the marmoset isolate (20P167W). The marmoset isolate possessed multiple virulence genes: *mrk* gene cluster (type III fimbriae) which is associated with cell entry and adhesion, *iro* and *iuc* gene cluster (salmochelin and aerobactin, respectively) associated with an iron-scavenging process (Figure 4). The marmoset genome data was subjected to antimicrobial-resistance genes using *in-silico* analysis with ResFinder 3.0 (28) on the Center for Genomic Epidemiology servers (http://www.genomicepidemiology.org/). We detected *bla*_*SHV*−67_, *bla*_*SHV*−11_, *oqxA, oqxB*, and *fosA* antimicrobial resistance genes.

### Phylogenetic tree

**Figure 5:**
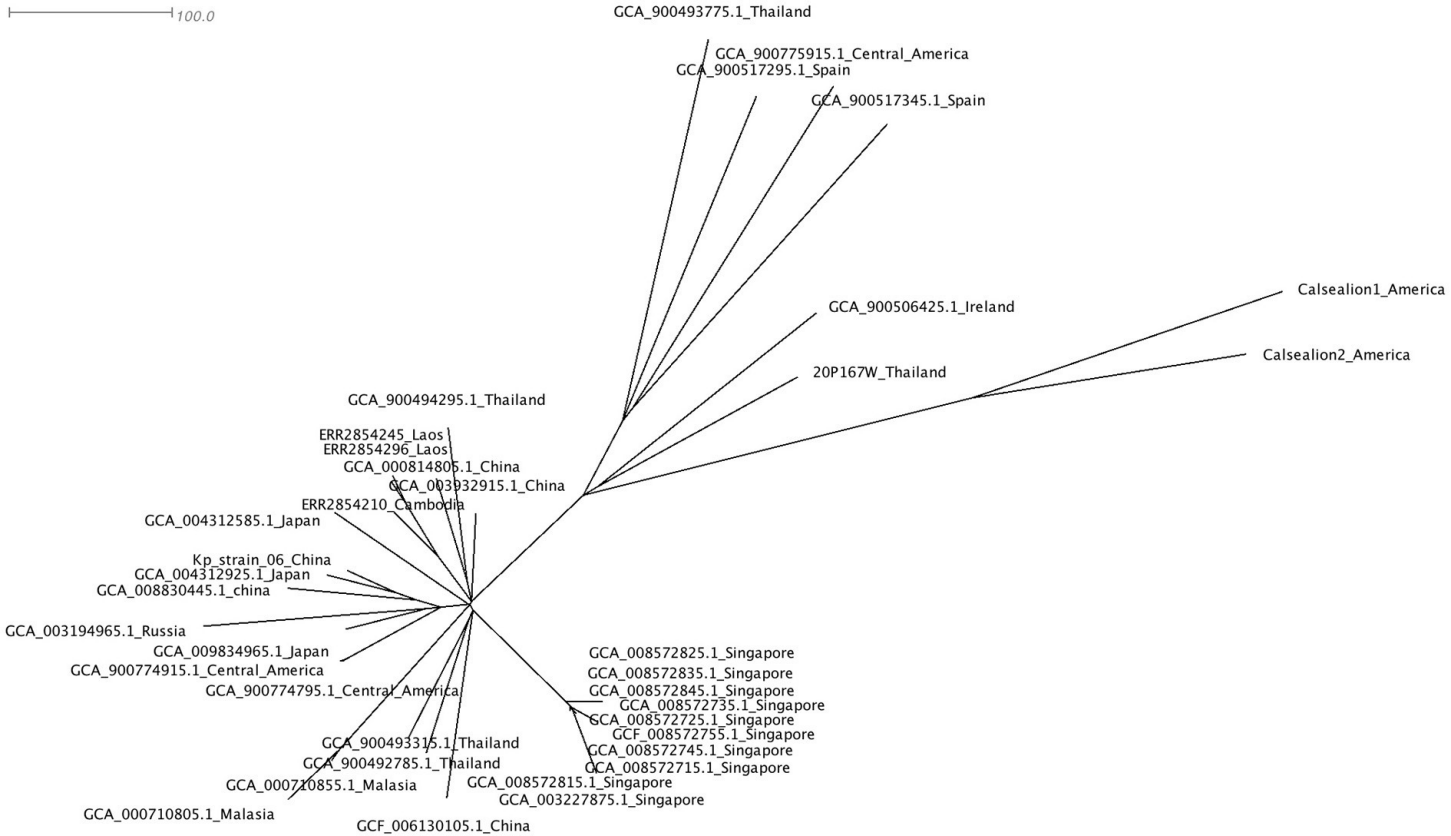
Data of virulence and antimicrobial resistance genes of each strain: rectangular represented *rmpA* and *rmpA2* genes, siderophore gene cluster including yersiniabactin, aerobactin, and salmochelin, fimbriae gene cluster, colibactin gene cluster, and antimicrobial resistance genes.

The cluster results showed that the marmoset isolate (20P167W) from Thailand was grouped together with the human isolates from Ireland (Figure 5). The marmoset isolate (20P167W) from Thailand was grouped separately from human isolates from Thailand and California sea lion isolates from America.

## Discussion and conclusions

Hypervirulent *K. pneumoniae* in humans causing invasive infections in both healthy and immunocompromised patients has been reported worldwide. This pathogen was also reported in animals causing fatal infection. In this fatal infection cases, all the isolates from four animals revealed *K. pneumoniae* with similar characters including serotype K2, positive *rmpA* gene (by PCR), positive string test and intermediate susceptible to amoxycillin/clavulanic acid, suggesting that this infection originated from a single clonal clone of bacterium. In human, hypervirulent *K. pneumoniae* are mostly associated with capsular serotype K1 and K2, which capsular serotype K2 was also found in our study. A previous case in marmosets from Brazil as well as other animals including New Zealand sea lions (10), California sea lions (12), southern sea otters (11) also had serotype K2. In comparison with serotype K1, serotype K2 has more genetic diversity. In human cases in Asia, sequence type 86 (ST86) and 65 (ST65) are the most prevalent among the serotype K2 group (29), which is similar to what we found in this study. The isolates from California sea lion, and marmoset in our study are ST65, while others (isolates from New Zealand sea lions and the isolate from marmoset from Brazil) are ST86 (15, 30). These can be implied that there is a genetic variation of hypervirulent *K. pneumoniae* in animals, suggesting that there is no specific genetic type for each species of animals.

In humans, liver abscesses are the main clinical sign of hypervirulent *K. pneumoniae* frequently complicated with septicaemia and spread to the central nervous system (1). In animals, liver abscess was rarely reported. There is only one case from a California sea lion (12). In marmosets, both reports had acute septicaemia and bacteria were seen in multiple organs as well as in the central nervous system (15). Pleuritis and suppurative pneumonia were seen in the California sea lion (12). Septicaemia and meningitis were reported in New Zealand sea lion pups (10). The difference of disease presentation in humans and animals may be due to several reasons, including host immune response, bacterial genetics, duration of infection, and route of infection. Based on human research, it is reported that the bacteria originate from the gut, and the liver may be the first target organ of invasion (1). However, the mechanism of pathogen translocation from the gut to liver is still unclear and needs further investigation (31). In captive and wild African green monkeys, HMV *K. pneumoniae* were found in rectal swabs, which indicate the colonization of this bacteria in the gut (13). Unfortunately, the study of the virulence of these isolates in African Green monkeys was undone. It is possible that the HMV *K. pneumoniae* isolated may be non-virulent strains and may colonize the gut as normal flora. Interestingly, in both humans and animals, hypervirulent *K. pneumoniae* can invade the central nervous system, which is uncommon for enteric gram-negative bacilli (3), suggesting the virulence of this pathogen in a wide range of hosts.

The genomic study showed that the marmoset isolate (20P167W) had similar genome size to other *K. pneumoniae* isolates with size of around 5- 6 Mbp (32, 33). The marmoset isolate possessed multiple virulence genes: *rmp*A and *rmp*A2, which associated with hypermucoviscous phenotype, *mrk* gene cluster (type III fimbriae) which is associated with cell entry and adhesion, and the *iro* and *iuc* gene cluster (salmochelin and aerobactin, respectively) associated with an iron-scavenging process. This correlates with other studies showing that HV *K. pneumoniae* possessed accessory siderophores rather than enterobactin, which is the core siderophore in Enterobacteriaceae bacteria (7). This data supports the theory that the iron up-take associated genes are one of the virulence factors in *K. pneumoniae* (31, 32). Compared with other human ST65 isolates that cause serious infection, the marmoset isolate possesses less number of virulence genes, but cause acute septicaemia in the marmosets. This brings about the possibility that the dead marmosets might have been immune suppressive.

We detected *bla*_*SHV*−67_, *bla*_*SHV*−11_, *oqxA, oqxB*, and *fosA* antimicrobial resistance genes. The *bla*_SHV−11_ gene (Figure 4) may be related to the intermediate resistance to amoxycillin/clavulanic acid. Normally, the strains that possess *bla*_SHV−11_ are susceptible to amoxycillin/clavulanic acid, but overproduction of SHV-11 β-lactamases may increase resistance to amoxycillin/clavulanic acid, similar to what was reported in *bla*_SHV−1_ gene (34). Generally, hypervirulent clones are susceptible to antibiotics, however, the genome study showed that a number of ST65 isolates possess several mobile antibiotic resistance genes, particularly in Asian countries where antibiotics are often misused. The increase of hypervirulent strains with antibiotic resistance genes should be recognized worldwide. This also suggests that monitoring of *K. pneumoniae* ST65 isolates should be continued worldwide in both human and animals. Notably, all of *K. pnuemoniae* ST65 isolates in both human and animal possess *bla*_*SHV*−67_, *bla*__*SHV*−11__, and *fosA* genes which is different from *K. pnuemoniae* ST86 (15).

In pan and core genome study, the number of unique genes increased when more isolates were added, suggesting that *K. pneumoniae* ST65 groups have an open genome. This raises a major concern that this group is able to accept genetic mobile elements such as antimicrobial resistance genes as well as other significant virulence genes, which leads to a consequence of treatment difficulties.

A phylogenetic tree using core single-nucleotide polymorphisms (SNPs) (*n* = 37) showed that there was no genetic distinction between human and animal isolates suggesting the possibility of pathogen transmission between humans and animals. The marmoset isolate in this study shows close genetic relation to the human isolate from Ireland. The marmoset isolate (20P167W) from Thailand was grouped separately from human isolates from Thailand. This suggests that multiple clones of ST65 are distributed in Thailand. In addition, there is no geographic distribution related to the genetic type worldwide.

This study in marmosets increases awareness of the fact that there are HV *Klebsiella pneumoniae* ST65 isolates in Thailand, not only in human cases, but also in animals. There is a chance that animals can be a reservoir of this pathogen. On the other hand, there is a possibility that animals can acquire this pathogen from humans. However, in this study, we cannot confirm the origin of the pathogen identified from the marmosets, because there have been no studies conducted with humans that live in close proximity to these animals as well as the possible environmental and/or food contamination sources.

This study provides important baseline data to monitor HV *K. pneumoniae* in both humans and animals and, to the best extent, develop control procedures for this infection.

## Data availability statement

The datasets presented in this study can be found in online repositories. The names of the repository/repositories and accession number(s) can be found in the article/Supplementary material.

## Ethics statement

Ethical review and approval was not required for the animal study because the samples in this study were collected as part of routine necropsy examinations held at Chulalongkorn small animal hospital, Bangkok, Thailand.

## Author contributions

KP and NP conceived the ideas. WB, RT, and KC performed a necropsy and collected samples. KP, NB, and PB performed the laboratory analyses. KP and WB performed data analyses and led the writing of the manuscript. All authors contributed to the drafts and gave final approval for publication.

## Funding

This research was supported by the Wildlife Exotic and Aquatic Pathology Research Unit (WEAP RU), the Second Century Fund (C2F), and Chulalongkorn University and Mubadala Petroleum (Thailand). The funders had no role in study design, data collection and analysis, decision to publish, or preparation of the manuscript.

## Conflict of interest

The authors declare that the research was conducted in the absence of any commercial or financial relationships that could be construed as a potential conflict of interest.

## Publisher's note

All claims expressed in this article are solely those of the authors and do not necessarily represent those of their affiliated organizations, or those of the publisher, the editors and the reviewers. Any product that may be evaluated in this article, or claim that may be made by its manufacturer, is not guaranteed or endorsed by the publisher.
