# Supplementary material for: Genetic characterization of hypervirulent Klebsiella pneumoniae responsible for acute death in captive marmosets
Source: Front Vet Sci. 2022 Aug 4;9:940912. doi: 10.3389/fvets.2022.940912 (PMC9397405; doi:10.3389/fvets.2022.940912)
Supplement: Supplementary file 1 [file Table_1.DOCX]

Supplement data

Genome data used in this article

| Strain Name | Source Host | Reference |
| --- | --- | --- |
| GCF 008572755.1 Singapore | Human | [Klebsiella pneumoniae genome assembly ASM857275v1](https://www.ncbi.nlm.nih.gov/assembly/4660021) |
| GCA 008572715.1 Singapore | Human | [Klebsiella pneumoniae genome assembly ASM857271v1](https://www.ncbi.nlm.nih.gov/assembly/4659991) |
| GCA 008572745.1 Singapore | Human | [Klebsiella pneumoniae genome assembly ASM857274v1](https://www.ncbi.nlm.nih.gov/assembly/4660001) |
| GCA 008572725.1 Singapore | Human | [Klebsiella pneumoniae genome assembly ASM857272v1](https://www.ncbi.nlm.nih.gov/assembly/4659981) |
| GCA 008572825.1 Singapore | Human | [Klebsiella pneumoniae genome assembly ASM857282v1](https://www.ncbi.nlm.nih.gov/assembly/4660051) |
| GCA 003227875.1 Singapore | Human | [Klebsiella pneumoniae genome assembly ASM322787v1](https://www.ncbi.nlm.nih.gov/assembly/1752891) |
| GCA 008572835.1 Singapore | Human | [Klebsiella pneumoniae genome assembly ASM857283v1](https://www.ncbi.nlm.nih.gov/assembly/4660031) |
| GCA 008572815.1 Singapore | Human | [Klebsiella pneumoniae genome assembly ASM857281v1](https://www.ncbi.nlm.nih.gov/assembly/4660041) |
| GCA 008572845.1 Singapore | Human | [Klebsiella pneumoniae genome assembly ASM857284v1](https://www.ncbi.nlm.nih.gov/assembly/4660061) |
| GCA 900493315.1 Thailand | Human | [Klebsiella pneumoniae genome assembly 22036_6#62](https://www.ncbi.nlm.nih.gov/assembly/1830561) |
| GCA 900492785.1 Thailand | Human | [Klebsiella pneumoniae genome assembly 22036_6#19](https://www.ncbi.nlm.nih.gov/assembly/1829271) |
| GCF 006130105.1 China | Human | [Klebsiella pneumoniae genome assembly ASM613010v1](https://www.ncbi.nlm.nih.gov/assembly/3338661) |
| GCA 004312925.1 Japan | Human | [Klebsiella pneumoniae genome assembly ASM431292v1](https://www.ncbi.nlm.nih.gov/assembly/2328841) |
| Kp strain 06 China | Human | [Klebsiella pneumoniae genome assembly ASM1342072v1](https://www.ncbi.nlm.nih.gov/assembly/7361551) |
| GCA 008830445.1 China | Human | [Klebsiella pneumoniae genome assembly ASM883044v1](https://www.ncbi.nlm.nih.gov/assembly/4790551) |
| GCA 009834965.1 Japan | Human | [Klebsiella pneumoniae genome assembly ASM983496v1](https://www.ncbi.nlm.nih.gov/assembly/5435691) |
| GCA 003194965.1 Russia | Human | [Klebsiella pneumoniae genome assembly ASM319496v1](https://www.ncbi.nlm.nih.gov/assembly/1736171) |
| GCA 900774795.1 Central America | Human | [Klebsiella pneumoniae genome assembly 25293_8#19-2](https://www.ncbi.nlm.nih.gov/assembly/2557701) |
| GCA 900774915.1 Central America | Human | [Klebsiella pneumoniae genome assembly 25293_8#25-2](https://www.ncbi.nlm.nih.gov/assembly/2557821) |
| GCA 004312585.1 Japan | Human | [Klebsiella pneumoniae genome assembly ASM431258v1](https://www.ncbi.nlm.nih.gov/assembly/2328641) |
| GCA 000710855.1 Malasia | Human | [Klebsiella pneumoniae T2-1-1 genome assembly De novo Assembly](https://www.ncbi.nlm.nih.gov/assembly/188551) |
| GCA 000710805.1 Malasia | Human | [Klebsiella pneumoniae T2-1-2 genome assembly De novo Assembly](https://www.ncbi.nlm.nih.gov/assembly/188531) |
| ERR2854245 Laos | Human | Wyres, Nguyen, et al., 2020 |
| ERR2854296 Laos | Human | Wyres, Nguyen, et al., 2020 |
| ERR2854210 Cambodia | Human | Wyres, Nguyen, et al., 2020 |
| GCA 000814805.1 China | Human | [Klebsiella pneumoniae subsp. pneumoniae 1158 genome assembly ASM81480v1](https://www.ncbi.nlm.nih.gov/assembly/240991) |
| GCA 900494295.1 Thailand | Human | [Klebsiella pneumoniae genome assembly 22036_6#356](https://www.ncbi.nlm.nih.gov/assembly/1830441) |
| GCA 003932915.1 China | Human | [Klebsiella pneumoniae genome assembly ASM393291v1](https://www.ncbi.nlm.nih.gov/assembly/2161771) |
| GCA 900775915.1 Central America | Human | [Klebsiella pneumoniae genome assembly 27097_7#118-2](https://www.ncbi.nlm.nih.gov/assembly/2558841) |
| GCA 900517345.1 Spain | Human | [Klebsiella pneumoniae genome assembly 18489_5#126](https://www.ncbi.nlm.nih.gov/assembly/1893561) |
| GCA 900517295.1 Spain | Human | [Klebsiella pneumoniae genome assembly 18489_5#122](https://www.ncbi.nlm.nih.gov/assembly/1893511) |
| GCA 900493775.1 Thailand | Human | [Klebsiella pneumoniae genome assembly 22036_6#279](https://www.ncbi.nlm.nih.gov/assembly/1829691) |
| GCA 900506425.1 Ireland | Human | [Klebsiella pneumoniae genome assembly 19506_1#134](https://www.ncbi.nlm.nih.gov/assembly/1899831) |
| 20P167W Thailand | Marmoset | This study |
| Calsealion1 America | California sea lion | Holt et al., 2015 |
| Calsealion2 America | California sea lion | Holt et al., 2015 |
